# Development of a hybrid LSTM with chimp optimization algorithm for the pressure ventilator prediction

**DOI:** 10.1038/s41598-023-47837-8

**Published:** 2023-11-27

**Authors:** Fatma Refaat Ahmed, Samira Ahmed Alsenany, Sally Mohammed Farghaly Abdelaliem, Mohanad A. Deif

**Affiliations:** 1 Department of Nursing, College of Health Sciences, University of Sharjah, Sharjah, UAE; 2 Critical Care and Emergency Nursing Department, Faculty of Nursing, Alexandria University, Alexandria, Egypt; 3 Department of Community Health Nursing, College of Nursing, Princess Nourah bint Abdulrahman University, P.O. Box 84428, 11671 Riyadh, Saudi Arabia; 4 Department of Nursing Management and Education, College of Nursing, Princess Nourah bint Abdulrahman University, P.O. Box 84428, 11671 Riyadh, Saudi Arabia; 5 Department of Artificial Intelligence, College of Information Technology, Misr University for Science and Technology (MUST), 6th of October City, 12566 Egypt

**Keywords:** Biotechnology, Engineering, Biomedical engineering

## Abstract

The utilization of mechanical ventilation is of utmost importance in the management of individuals afflicted with severe pulmonary conditions. During periods of a pandemic, it becomes imperative to build ventilators that possess the capability to autonomously adapt parameters over the course of treatment. In order to fulfil this requirement, a research investigation was undertaken with the aim of forecasting the magnitude of pressure applied on the patient by the ventilator. The aforementioned forecast was derived from a comprehensive analysis of many variables, including the ventilator's characteristics and the patient's medical state. This analysis was conducted utilizing a sophisticated computational model referred to as Long Short-Term Memory (LSTM). To enhance the predictive accuracy of the LSTM model, the researchers utilized the Chimp Optimization method (ChoA) method. The integration of LSTM and ChoA led to the development of the LSTM-ChoA model, which successfully tackled the issue of hyperparameter selection for the LSTM model. The experimental results revealed that the LSTM-ChoA model exhibited superior performance compared to alternative optimization algorithms, namely whale grey wolf optimizer (GWO), optimization algorithm (WOA), and particle swarm optimization (PSO). Additionally, the LSTM-ChoA model outperformed regression models, including K-nearest neighbor (KNN) Regressor, Random and Forest (RF) Regressor, and Support Vector Machine (SVM) Regressor, in accurately predicting ventilator pressure. The findings indicate that the suggested predictive model, LSTM-ChoA, demonstrates a reduced mean square error (MSE) value. Specifically, when comparing ChoA with GWO, the MSE fell by around 14.8%. Furthermore, when comparing ChoA with PSO and WOA, the MSE decreased by approximately 60%. Additionally, the analysis of variance (ANOVA) findings revealed that the p-value for the LSTM-ChoA model was 0.000, which is less than the predetermined significance level of 0.05. This indicates that the results of the LSTM-ChoA model are statistically significant.

## Introduction

### Background

Numerous illnesses call for a ventilator in medicine and medical transportation systems. The poliomyelitis pandemic in the 1950s provided conclusive evidence of a mechanical ventilator's effectiveness^1–3^. The ventilator can assist in moving air into and out of the lungs if a person has respiratory failure, hypoxemia, or hypercapnia and cannot breathe properly^4,5^. Furthermore, if someone has surgery under general anesthesia, they will also need a ventilator for adequate breathing. However, because mechanical ventilators are time-consuming, expensive, and ineffective, it is particularly challenging to have them available for all patients during a pandemic. Machine learning, on the other hand, can support autonomous pressure selection and prediction^6^. Many traditional mechanical ventilator prediction techniques have been introduced in recent years. Nonetheless, they should have paid more attention to the costly newly developed technologies and correct pressure because a doctor manually adjusted them^7^.

Combining derivative, integral, and proportional controls is the industry standard for proportional-integral-derivative (PID) controllers. In this fusion, the discrepancy from the desired waveform is employed as input, leading to adjustments in the pressure chamber to rectify the output waveform and minimize the margin of deviation. Pressure inaccuracies emerge from this operating design, which calls for continual human surveillance and intervention by medical experts. This method complies with the required requirements and safety standards; however, it lacks the adaptability and quickness needed for a variety of clinical scenarios. Therefore, this problem might be solved with a dynamic controller that can continuously adjust pressure settings. This is the precise situation where replacing the traditional PID controller algorithm with machine learning comes into play.

Due to the recent COVID-19 pandemic, a considerable array of open-source ventilator designs has emerged. This situation has paved the way for numerous endeavors to enhance these ventilators using advanced techniques and systems. These efforts involve incorporating decision support methods reliant on machine learning models that are finely tuned to incorporate patient responses. The overarching aim is to enhance the accuracy of pressure values.

There have been more and more studies on the forecasting of ventilator pressure using LSTM networks because it has been shown that the LSTM, as a type of Recurrent Neural Network (RNN)^8,9^, is suited for evaluating long-series data. The relevant hyperparameters must be changed to build an LSTM network model that satisfies the specifications^10^, and researchers frequently choose these values based on past knowledge and experience^11^. It could be necessary to adjust the pertinent parameters for specific problems manually^12,13^.

### Related works and problem statement

Due to the increasing complexity and multidimensionality of almost every aspect of the real world, research into optimization problems remains robust. The optimization process involves determining the optimal configuration of variables based on the objective function of the optimization problem. However, traditional optimization methods sometimes struggle to provide timely solutions. Therefore, a many of nature-inspired metaheuristic algorithms have been developed to tackle challenging problems. These include genetic algorithm (GA)^14^, simulated annealing algorithm (SA)^15^, particle swarm optimization (PSO)^16^, ant colony optimization (ACO)^17^, artificial bee colony (ABC)^17^, differential evolution (DE)^18^, harmony search (HS)^19^, firefly algorithm (FA)^20^, bat algorithm (BA)^21^, flower pollination algorithm (FPA)^22^, dragonfly algorithm (DA)^23^, grey wolf optimizer (GWO)^24^, moth-flame optimization algorithm (MFO)^25^, earthworm optimization algorithm (EWA)^26^, elephant herding optimization (EHO)^27^, brain storm optimization algorithm (BSO)^28^, fireworks algorithm (FWA)^29^, moth search (MS)^30^, squirrel search algorithm (SSA)^31^, salp swarm algorithm (SSA)^32^ and whale optimization algorithm (WOA)^25^. Numerous studies have helped to advance the LSTM network optimization techniques in recent years. Table 1 provides information about the models that were used as well as a list of current related research.

**Table 1:** Provides information about the optimization models that were used in related research.

References	Optimization model	Research aim
^33^	Gradient clipping and weight initialization	Address the challenges of training LSTMs by proposing techniques to tackle the vanishing/exploding gradient problem
^34^	Proper weight initialization	The paper discussed the importance of appropriate weight initialization techniques in facilitating the training of deep neural networks, including LSTMs
^35^	Non-saturating activation functions and gradient clipping	The study explored the challenges associated with training RNNs, including LSTMs, and proposed techniques to address the vanishing/exploding gradient problem
^36^	Adam optimization algorithm	The authors introduced Adam as an efficient and effective optimization algorithm for training neural networks, including LSTMs, and demonstrated its benefits in handling different optimization problems
^37^	Scheduled sampling	The study introduced scheduled sampling as a technique for training sequence prediction models, including LSTMs. It addressed the discrepancy between training and inference by gradually replacing ground truth inputs with model predictions during training
^38^	Layer normalization	The internal covariate shift issue in deep neural networks, including LSTMs, was discussed in the study, and layer normalization was suggested as a solution. It sought to expedite training and stabilize optimization
^39^	Residual learning framework	The research introduced the residual learning framework, which allows for training very deep neural networks, including LSTMs, by using skip connections. It mitigated the vanishing/exploding gradient problem and enabled the training of deeper LSTM architectures
^40^	Stochastic gradient descent (SGD), mini-batch training, learning rate schedules, and adaptive optimization algorithms	The study provided an overview of optimization methods for large-scale machine learning tasks, including LSTM optimization. It discussed various techniques to optimize LSTMs and other models in the context of large-scale datasets and computational constraints
^41^	the modified bald eagle search (MBES) algorithm, PSO, GA, GWO	the select the LSTM hyperparameters using

Every optimization technique comes with its own set of advantages and disadvantages. The selection of an approach hinges on the particular demands of the task, the network's structure, and the computational resources at hand. It is often necessary to experiment and fine-tune these techniques to achieve optimal results for a given LSTM-based application.

1. Gradient clipping and weight initialization:
  Benefits: Gradient clipping helps address the vanishing/exploding gradient problem by imposing a threshold on the gradients during training. It prevents gradients from becoming too large or too small, leading to more stable and effective training. Proper weight initialization techniques, such as Glorot and He initialization, ensure that the weights are initialized in a way that promotes efficient gradient flow and convergence.
  Drawbacks: Gradient clipping can potentially introduce biases to the gradient updates and may require careful tuning of the threshold value. Weight initialization techniques may not always guarantee the optimal initialization for all network architectures or tasks, and finding the appropriate initialization scheme can still be a trial-and-error process.
2. Non-saturating activation functions and gradient clipping:
  Benefits: Non-saturating activation functions, such as ReLU (Rectified Linear Unit), help mitigate the vanishing gradient problem by avoiding saturation in the activation values. They facilitate the flow of gradients and enable better learning in deep architectures. Gradient clipping, as mentioned earlier, prevents gradients from becoming too large, ensuring more stable training.
  Drawbacks: Activation functions such as ReLU, which do not saturate, can encounter the issue of the "dying ReLU" phenomenon. This occurs when certain neurons become inactive and fail to recuperate throughout the training process. This issue can hinder the learning process and network performance. Gradient clipping, if applied too aggressively, can result in underutilization of the gradient information and slow convergence.
3. Adam optimization:
  Benefits: Adam combines adaptive learning rates and momentum to efficiently optimize LSTM networks. By adjusting the learning rate on a per-parameter basis, it achieves enhanced optimization performance and quicker convergence. Adam also maintains separate learning rates for each parameter, making it suitable for dealing with sparse gradients and noisy data.
  Drawbacks: Adam has several hyperparameters that require careful tuning, such as the learning rate, momentum parameters, and exponential decay rates. Poor hyperparameter selection can lead to suboptimal results or difficulties in convergence. Additionally, Adam may not always generalize well to all types of optimization problems and could benefit from modifications in certain scenarios.
4. Scheduled sampling:
  Benefits: Scheduled sampling addresses the discrepancy between training and inference by gradually introducing model predictions during training. It allows the model to adapt to the errors made by its own predictions, leading to improved performance during inference. This technique is particularly useful in sequence prediction tasks, where the model needs to generate accurate outputs based on its own predictions.
  Drawbacks: Scheduled sampling introduces a discrepancy between the training and inference processes, which can make the optimization task more challenging. Determining the appropriate schedule for introducing model predictions requires careful consideration and may vary depending on the specific task and dataset.
5. Layer normalization:
  Benefits: Layer normalization addresses the internal covariate shift problem by normalizing the inputs to each layer. It helps stabilize the optimization process, accelerates training, and allows for faster convergence. Layer normalization also provides better generalization and performance, particularly in deep networks.
  Drawbacks: Layer normalization introduces additional computational overhead, as it requires calculating the mean and variance of each layer's inputs. This overhead can make training slower compared to other normalization techniques. Additionally, the benefits of layer normalization may vary depending on the specific architecture and task, and it might not be universally applicable in all cases.
6. Residual learning:
  Benefits: Residual learning, achieved through skip connections, allows for training very deep networks, including LSTMs. It mitigates the vanishing/exploding gradient problem by providing shortcut connections that facilitate gradient flow. Residual connections enable the training of deeper LSTM architectures, leading to improved accuracy and better utilization of network depth.
  Drawbacks: The main challenge with residual learning is designing appropriate skip connections and ensuring that the information flow through the network is optimized. Poorly designed skip connections or overly deep architectures can still suffer from optimization difficulties. Additionally, residual learning may require careful tuning of hyperparameters and architectural decisions.

### Motivations, contributions, and article organization

In order to address these challenges in our study, the parameters of the LSTM model are fine-tuned using the ChoA algorithm, resulting in the development of the LSTM-ChoA prediction model. The experimental findings demonstrate that this optimized model effectively predicts ventilator pressure for patients, enabling mechanical ventilators to provide tailored patient care. The key contributions of this research can be summarized as follows:

1. The ventilator dataset was explored, analyzed, and added new features.
2. The ChoA algorithm optimized the parameters of the LSTM, and the ChoA -LSTM forecasting model was subsequently constructed.
3. ChoA algorithm's efficiency was compared with other metaheuristic optimization algorithms.
4. The prediction efficiency of LSTM-ChoA was compared with other regressor models.

This research article comprises several sections, namely: related works, inspiration, study objectives, theoretical framework and approach, experimentation, experimental results, and conclusion. The theoretical and methodological aspects encompass two key components: an explanation of the LSTM model theory, the ChoA algorithm, and a concise overview of our proposed methodology.

## Materials and methods

### Materials

**Figure 1:**
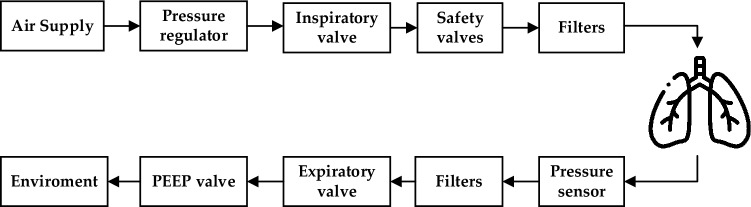
The diagram of the respiratory circuit.

The ventilator data used in this work was obtained from the Google Brain public dataset, which is publicly accessible on Kaggle^42^. An artificial bellows test lung was attached to a custom open-source ventilator using a respiratory circuit to create the dataset. Figure 1 shows the configuration, with the predicted state variable (airway pressure) indicated in blue and the two control inputs highlighted in green. The inspiratory solenoid valve's percentage of opening, which allows air into the lung, is represented by the first control input. It has a scale of 0–100, with 0 signifying total closure (no air allowed in) and 100 signifying complete opening. A binary variable showing whether the exploratory valve is open (1) or closed (0), allowing air to be discharged, serves as the second control input. The goal of positive end-expiratory pressure (PEEP) is to keep the lungs from collapsing^43^.

Each time series corresponds to a breath that lasts about three seconds. About 75,000 for training and 50,000 test breaths comprise the dataset's five features. Information on significant features and intended columns is compiled in Table 2.

**Table 2:** Description of features of the dataset.

Feature name	Feature abbreviation (unit)	Feature description
Resistance	R (cmH2O/L/S)	Restriction of the airway
Compliance	C (mL/cmH_2_O)	The ability of the lungs to stretch and expand
Time-step	Time-step	The actual time stamps
Inspiratory Solenoid Valve	ISV	Manipulation command for the inhalation solenoid valve
Exploratory Solenoid Valve	ESV	The input utilized for controlling the exploratory solenoid valve
Airway pressure	P (cmH2O)	The recorded airway pressure within the respiratory circuit is displayed in the designated column.

Among the observed values, R equal to 20 and C were found to be the most common in patients with severe lung disease^10^. The extent of openness of the inspiratory solenoid valve (ISV) is quantified as a continuous parameter spanning from 0 to 100. Here, 0 signifies a fully closed position, while 100 corresponds to complete openness. Alternatively, the exploratory valve status (ESV) serves as a binary variable denoting whether the valve is in the open state (1) or the closed state (0), facilitating air release.

#### Preliminary knowledge

##### Long short-term memory (LSTM)

**Figure 2:**
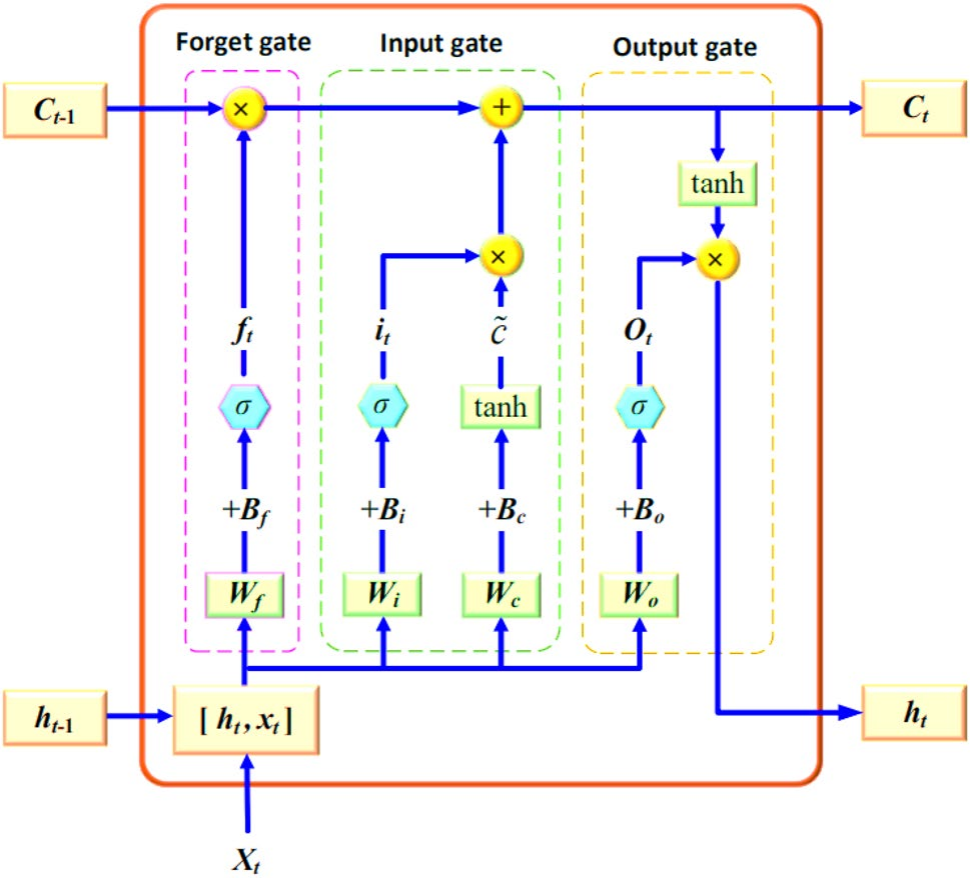
LSTM model structure.

The construction of the LSTM, a particular kind of RNN^44,45^, is depicted in Fig. 2. Its hidden layer comprises one or more memory cells, each with an input, output, and forget gate^46,47^.

**The forget gate** (${f}_{t}$): is responsible for deciding whether to retain the long-term state $c$ and is computed using the current time step *t* input ${x}_{t}$ and the output ${h}_{t-1}$ from the preceding time step $t-1$. The corresponding formula for this operation is:

1 $${f}_{t}=\sigma \left({W}_{f}\times \left[{h}_{t-1},{x}_{t}\right]+{B}_{f}\right)$$

The matrix ${W}_{f}$ corresponds to the weights linked with the forget gate, while ${B}_{f}$ denotes the biased term. The sigmoid function $\sigma (\cdot )$ is applied to the combined inputs to compute the forget gate's decision.

**The input gate's** is responsible for generating a new candidate cell state $\tilde{c}$, performing relevant computations, and controlling the amount of information to be added. The calculation formula for the input gate is as follows:

2 $${i}_{t}=\sigma \left({W}_{i}\times \left[{h}_{t-1},{x}_{t}\right]+{B}_{i}\right)$$

3 $${\widetilde{c}}_{t}=\mathrm{tanh}\left({W}_{c}\times \left[{h}_{t-1},{x}_{t}\right]+{B}_{c}\right)$$

In the given equation, the current input cell state represented by *'c'*, while ${W}_{c}$ corresponds to the weight matrix linked to the cell state, and ${B}_{c}$ signifies the bias term associated with the cell state. Similarly, ${i}_{t}$ represents the output generated by the input gate, ${W}_{i}$ is indicative of the weight matrix for the input gate, and ${B}_{i}$ stands for the bias term pertaining to the input gate. The matrix denoted as $\left[{h}_{t-1},{x}_{t}\right]$ encompasses two vectors: ${x}_{t}$, which signifies the input at the present time step (t), and ${h}_{t-1}$, representing the output from the prior time step (t-1). The sigmoid activation function is denoted by $\sigma (\cdot )$, while the hyperbolic tangent function is represented as $\mathrm{tanh}\left(\cdot \right)$.

**Output gate:** produces the output ${f}_{t}$ using a sigmoid function $\sigma (\cdot)$ that takes the input ${x}_{t}$ at the current time step ($t$ t) and the output ${h}_{t-1}$ from the previous time step ($t-1$) as inputs. The calculation can be expressed as follows:

4 $${O}_{t}=\sigma \left({W}_{o}\times \left[{h}_{t-1},{x}_{t}\right]+{B}_{o}\right)$$

5 $${c}_{t}={f}_{t}\times {c}_{t-1}+{i}_{t}\circ {\widetilde{c}}_{t}$$

6 $${h}_{t}={o}_{t}\times \mathrm{tanh}\left({c}_{t}\right)$$

${B}_{o}$ represents the output gate's bias term, and ${W}_{o}$ is the weight of the output gates.

The LSTM structure, with its unique three-gate architecture and hidden state with memory capabilities, effectively addresses the issue of long-term dependencies by capturing long-term historical information. The hidden state undergoes a series of operations. Firstly, the forget gate ${C}_{t}$ at the current time step (t) regulates which information from the previous hidden state ${C}_{t-1}$ at time step $t-1$ needs to be discarded and which information should be retained. Secondly, the structure of the hidden state selectively discards certain data while utilizing the input gate to incorporate new information in conjunction with the forget gate. Finally, the cell state ${C}_{t}$ is updated through a sequence of computations. The LSTM employs the output gate, the cell state ${C}_{t}$, and a tanh layer to calculate the final output value ${h}_{t}$.

The choice of the Long Short-Term Memory (LSTM) model for predicting ventilator pressure in this study likely stems from several advantages that make LSTMs well-suited for sequential data prediction. Here are some reasons why the authors might have chosen LSTM and its advantages^48–50^:

1. LSTMs are a type of recurrent neural network (RNN) designed to handle sequential data. Ventilator data is sequential in nature, as it consists of time-series measurements. LSTMs can capture dependencies over time, making them suitable for modeling such data.
2. LSTMs are designed to overcome the vanishing gradient problem, allowing them to capture long-term dependencies in data. In the context of ventilator data, where previous time steps can significantly impact future pressure values, LSTMs are capable of capturing these long-range dependencies.
3. LSTMs have an internal memory mechanism that allows them to retain information over long sequences. This is particularly useful in scenarios where previous states, such as lung capacity, play a crucial role in predicting future states, like airway pressure.
4. LSTMs are highly adaptable and can handle different input and output formats. This flexibility makes them suitable for a variety of machine learning tasks, including time series forecasting.
5. LSTMs can automatically learn relevant features from the data, reducing the need for extensive manual feature engineering. This can be advantageous when working with complex datasets like ventilator pressure data.
6. LSTMs can handle noisy data and are less sensitive to minor variations in the input data, which is beneficial when working with real-world data that may contain noise or measurement errors.
7. LSTMs are capable of capturing complex interactions between different features in the data. In the context of ventilator data, where various factors, such as the inspiratory solenoid valve and exploratory valve, can interact to affect airway pressure, this feature is valuable.
8. LSTMs can be effectively used for regression tasks, making them suitable for predicting numerical values like airway pressure.

It is important to note that while LSTMs offer these advantages, the model's success depends on factors such as data quality, hyperparameters' choice, and sufficient training data availability. The authors chose LSTM because of its ability to model sequential data effectively, suitability for time series forecasting tasks, and robustness in handling ventilator pressure data's complex and noisy nature.

##### Chimp optimization algorithm (ChoA)

**Figure 3:**
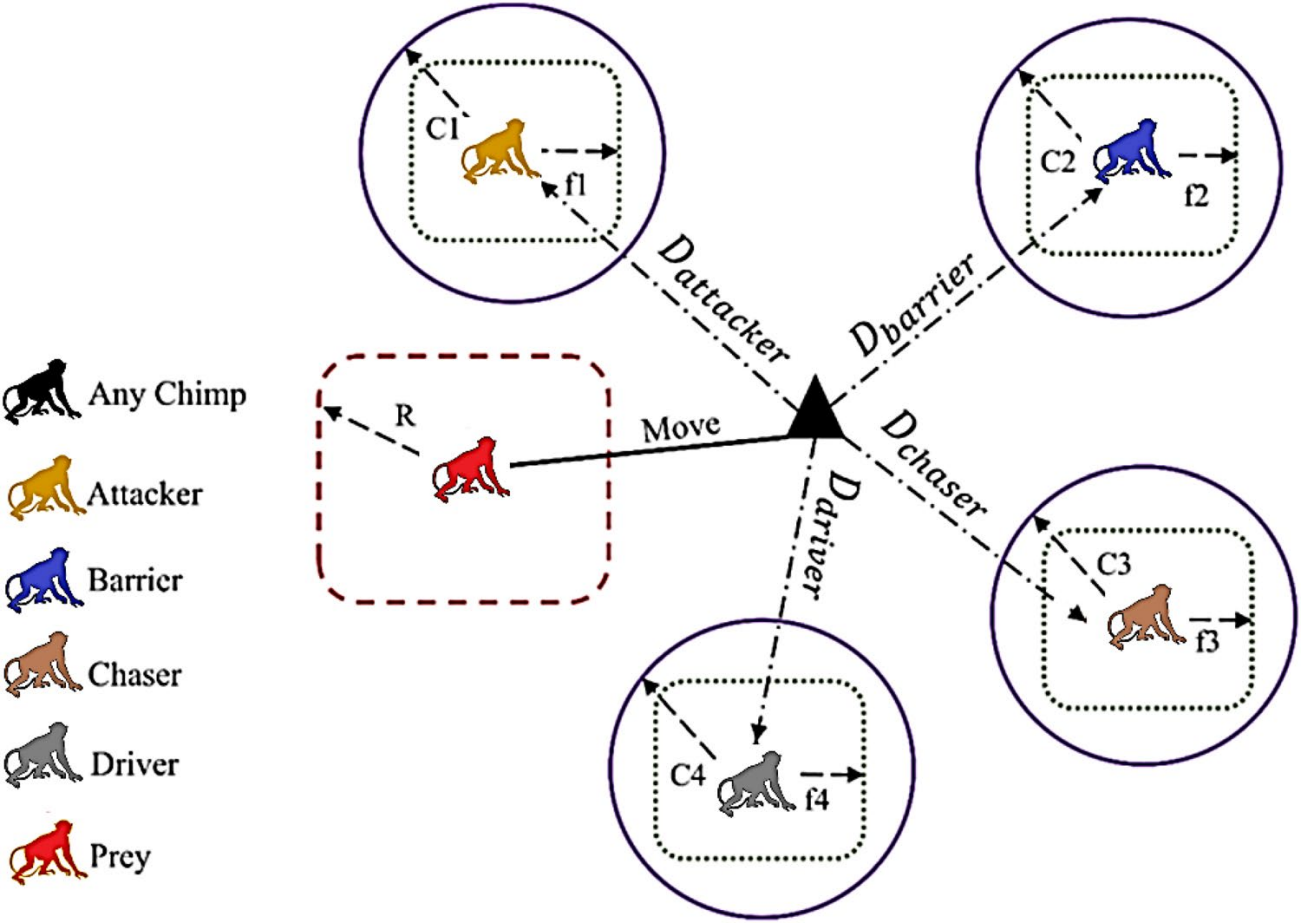
Updating the ChoA position.

This model draws inspiration from chimpanzee intelligence and breeding behavior in cluster hunting^51–53^. Figure 3 depicts attack, chase, barrier, and driver as the four methods used to replicate the attitude. Equation (7) represents the distance $(D)$ between the chimp and its prey. Equation (8) represents the chimpanzee’s position update formula; ${\alpha }_{\text{prey}}$ is a vector of prey positions; and ${\alpha }_{\text{chimp}}$ is a position vector for chimpanzees^54^.

7 $$D=\left|C\cdot {\alpha }_{{\text{prey}} \, }-m\cdot {\alpha }_{\text{chimp}}\right|$$

8 $${\alpha }_{{\text{chimp}} \, }(n+1)=\left|{\alpha }_{{\text{prey}} \, }-a\cdot d\right|$$

Where the co-efficient vectors are $m,c$, and $d$, these elements can be justified by (9) through (11).

9 $$a=2\cdot l\cdot {r}_{1}-1$$

10 $$c=2\cdot {r}_{2}$$

11 $$m= \, \text{chaotic value}$$

where $l$ is a constant that drops along a line from 2.5 to 0 throughout the iterations^55^, ${r}_{1}$ and ${r}_{2}$ are random values between 0 and 1, and m is a disordered vector. The attacker, barrier, chaser, and driver are the best outputs with optimal capabilities for mathematically simulating this system^56,57^. $C$ is a random variable that influences the position of prey within [0, 2] on the individual position of chimps (when $C$ < 1, the degree of influence weakens; when $C$ > 1, the degree of influence strengthens).

The positions of other chimps in the population are determined by the positions ($d$) of Attacker, Barrier, Chaser, and Driver, and the position update Eqs. (12), (13), (14), and (15)^58^.

12 $${d}_{{\text{attack}} \, }=\left|{C}_{1}\cdot {\alpha }_{{\text{attacker}} \, }-{m}_{1}\cdot {x}_{n}\right|$$

13 $${d}_{{\text{barrier}} \, }=\left|{C}_{2}\cdot {\alpha }_{{\text{barrier}} \, }-{m}_{2}\cdot {x}_{n}\right|$$

14 $${d}_{{\text{chaser}} \, }=\left|{C}_{3}\cdot {\alpha }_{{\text{chaser}} \, }-{m}_{3}\cdot {x}_{n}\right|$$

15 $${d}_{{\text{driver}} \, }=\left|{C}_{4}\cdot {\alpha }_{{\text{driver}} \, }-{m}_{4}\cdot {x}_{n}\right|$$

Where $\alpha$ is a positions vector of the four chimp. Following this, the chimpanzees' next point (${x}_{1}, {x}_{2,}{x}_{3} and {x}_{4}$)is reorganized using (16) through (19):

16 $${x}_{1}={\alpha }_{{\text{attacker}} \, }-{a}_{1}\cdot {d}_{\text{attacker}}$$

17 $${x}_{2}={\alpha }_{{\text{barrier}} \, }-{a}_{2}\cdot {d}_{\text{barrier}}$$

18 $${x}_{3}={\alpha }_{{\text{chaser}} \, }-{a}_{3}\cdot {d}_{\text{chaser}}$$

19 $${x}_{4}={\alpha }_{{\text{driver}} \, }-{a}_{4}\cdot {d}_{\text{driver}}$$

The positions are upgraded by using Eq. (20):

20 $${x}_{{\text{chimp}} \, }=\frac{{x}_{1}+{x}_{2}+{x}_{3}+{x}_{4}}{4}$$

Then, Eq. (21) is implemented once the positions have been upgraded.

21 $${\alpha }_{{\text{chimp}} \, }(n+1)=\left\{\begin{array}{ll}{\alpha }_{{\text{prey}} \, }-x\cdot d& ,\varnothing <0.5\\ \, {\text{chaotic}} \, & ,\varnothing <0.5\end{array}\right.$$

The motivation for using the Chimp Optimization Algorithm (ChoA) to optimize the feature space in our study can be explained as follows:

1. The feature space in machine learning models is often non-convex and high-dimensional, making it challenging to find the optimal combination of features manually. ChoA is designed to handle non-convex optimization problems, making it well-suited for feature selection and hyperparameter optimization.
2. ChoA can efficiently identify relevant features in the dataset, which is crucial for improving model performance. By selecting the most informative features, we aim to enhance the accuracy of our airway pressure prediction model while reducing the dimensionality of the input data.
3. ChoA is not limited to feature selection but can also optimize hyperparameters of machine learning models. In our study, ChoA is employed to fine-tune the hyperparameters of the Long Short-Term Memory (LSTM) model, which plays a crucial role in predicting airway pressure accurately.
4. The primary motivation behind using ChoA is to enhance the overall performance of our predictive model. By optimizing both the feature space and model hyperparameters, we aim to achieve superior accuracy and predictive capabilities in comparison to other optimization techniques.
5. The utilization of ChoA in the context of feature selection and hyperparameter optimization is relatively novel. By applying this innovative optimization approach, we intend to contribute to the scientific knowledge in the field of machine learning and healthcare by showcasing the potential benefits of ChoA in enhancing predictive models.

In summary, the motivation for using ChoA lies in its ability to address the challenges posed by complex feature spaces, efficiently select the most relevant features, fine-tune model hyperparameters, and ultimately improve the performance of our airway pressure prediction model. By employing ChoA, we aim to demonstrate its effectiveness and showcase its potential contributions to the field of healthcare and predictive modeling.

### Methodology

**Figure 4:**
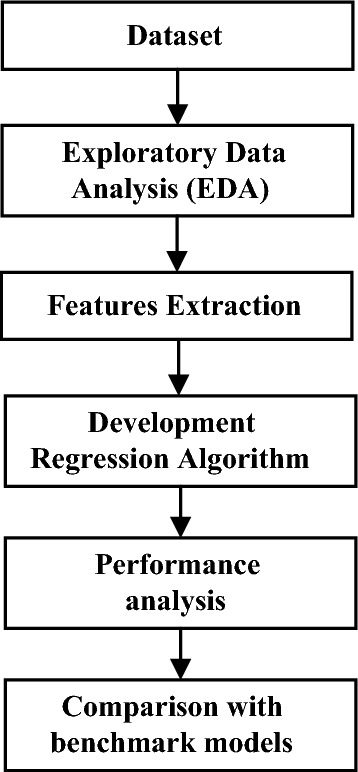
Overall methodology phases.

As shown in Figure 4, our proposed approach for ventilator pressure prediction includes five steps. The study encompassed several stages, starting with Exploratory Data Analysis (EDA) and progressing through feature extraction, the development of a regression model, an assessment of the model's performance, and a subsequent comparison of the outcomes achieved by the proposed regression model with those of benchmark models. Each phase is described in detail below.

#### Exploratory data analysis

This process enabled us to extract additional meaningful insights from the ventilator dataset,^36^ uncover distinct patterns within the data, and identify the most suitable models that utilize the available features to achieve faster convergence^61^ and minimize prediction errors^62^.

The exploratory Data Analysis involves studying the correlation between dataset variables and airway pressure patterns during the inhalation and exhalation phases. Finally, analysis of the Effects R–C pairs have on pressure distribution. Our study uses Spearman's rank correlation ($\rho$) to assess the correlation between dataset features as follows^63^:

22 $$\rho =1-\frac{6\sum {d}_{i}^{2}}{n\left({n}^{2}-1\right)}$$

where $n$ is the number of observations and ${d}_{i}$ is the difference between each observation.

#### Extracted features

Based on previous analysis, It was decided to create and extract four features that improve the model predictions and converge the model faster^64^. The following features were extracted:

1. Lung setting (δ) = R*C: It indicates the change in volume per change in flow
2. CISV is defined as the cumulative sum of the control input for the inspiratory solenoid valve.
3. Integral is the difference between consecutive time steps.
4. Differential = indicates the difference of consecutive samples.

#### Development regression algorithm

**Figure 5:**
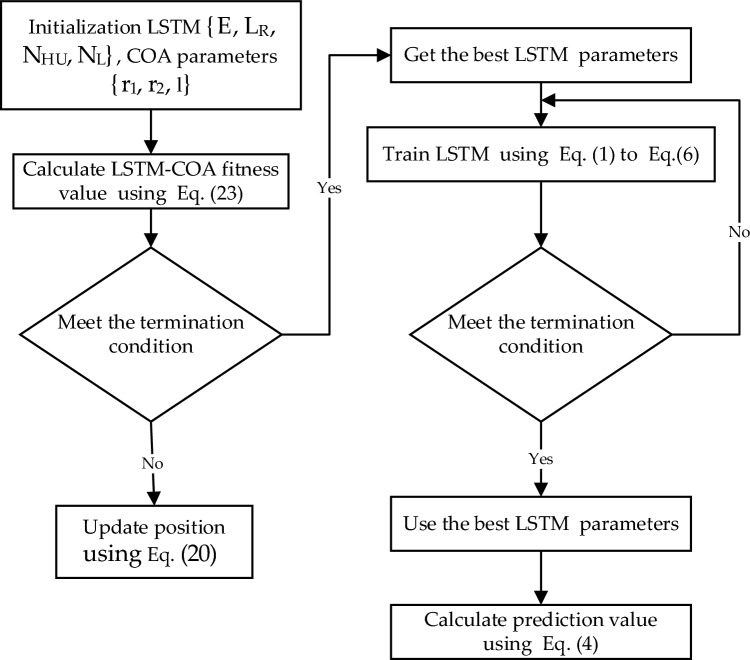
Flowchart of the LSTM- ChoA algorithm.

The process of selecting a set of hyperparameters that yield optimal performance for the Long Short-Term Memory (LSTM) model on the given data is referred to as Hyperparameter Optimization (HPO). In this work, the Chimp Optimization Algorithm (ChoA) is employed to determine the best values for the LSTM Model Hyperparameters. These hyperparameters include the Number of Iterations (N_I_), Learning Rate (L_R_), Hidden Units (NHU), and Number of Layers (N_L_). Figure 5 portrays the flow chart that presents the LSTM forecasting model known as the LSTM-ChoA model. The process encompasses distinct steps outlined below:

**Step (1):** Initialize LSTM {E, L_R_, N_HU_, N_L_} parameters and the ChoA algorithm parameters that include the chimp population $\left\{{r}_{1},{r}_{2},l\right\}$ and the constants $\left\{a,\mathrm{c},m\right\}$.

**Step (2):** The mean square error gained from training the LSTM network was used to compute the fitness function, which corresponds to the cost function value. This fitness value was continuously updated in real-time as the Chimp optimization algorithm progressed. Within the range of iterations, Eq. (9) was utilized to determine the position of each chimp, and subsequently, the chimps were randomly divided into independent groups.

The cost function is Mean Squared Error (MSE), which distinguishes between real and predicted data. The MSE function must be reduced during LSTM training by updating hyperparameter values. The MSE is presented in Eq. (23).

23 $$\mathrm{MSE}=\frac{\sum_{i=0}^{n} {\left(y-{y}^{\mathrm{^{\prime}}}\right)}^{2}}{n}$$

**Step (4):** Assign the best chimp in each group using Eqs. (12), (13), (14), and (15), respectively.

**Step (3):** If the current new position was better, the old position was updated using Eq. (20).

**Step (5):** The LSTM network was trained based on the optimal parameter combination, and testing samples were utilized for forecasting.

#### Benchmark models

The evaluation of ChoA's performance involved a comparison with several benchmark optimization methods, such as whale optimization algorithm (WOA), grey wolf optimizer (GWO)^65^, and particle swarm optimization (PSO)^66^. The initial parameter values for each optimizer can be found in Table 3.

**Table 3:** Parameter value of PSO, GWO, WOA, and ChoA.

Optimizer	Hyper-parameter name	Parameter value
Common setting	Population size	$\mathrm{P}=10$
	Maximum iteration	$\mathrm{N}=200$
	Runs	$\mathrm{r}=20$
	Probability	$\mathrm{Pr}=0.5$
PSO	Number of particles	S = 30
	Cognitive factor and social factor	C_1_ = C_2_ = 0.75
GWO	Population size	P_S_ = 10
	Gene mutation	G_M_ = 0.10
	Gene crossover	G_C_ = 0.5
	generations number	G_n_ = 1.5
WOA	Whales number	W_n_ =50
ChoA	Random vectors	in the range of [0, 1]
	Constant	$l$ = 2.5

Furthermore, the LSTM- ChoA algorithm has been compared with regression models as baselines: Random forest (RF) Regressor, Support Vector Machine (SVM) Regressor, and K-nearest neighbor (KNN) Regressor. The optimal Hyper-parameter for baseline models was selected using grid search in sklearn Libraries^59,60^. In Table 4, the search space for the hyperparameters of the regressor models is presented, along with the selected hyperparameters obtained through grid search.

**Table 4:** The range of potential values for the hyperparameters of the regressor models.

Model	Hyper-parameter	Search space	Selected Hyper-parameter
RF	n estimators	[10, 100]	36
	max depth	[5, 50]	6
	min samples leaf	[1, 11]	2
	min samples split	[2, 11]	6
	criterion	[' mse'' mae']	mse
	max features	[1, 13]	6
	C	[0.1, 50]	50
SVM	kernel	['linear', 'poly', 'rbf',' sigmoid']	rbf
	epsilon	[0.001, 1]	0.001
KNN	n neighbors	[1, 20]	5

### Experimental simulation configuration

The methodology introduced was put into practice using Jupyter Notebook version 6.5.4^67^ along with Python version 3.6.7^68^. The execution took place on a personal computer equipped with an Intel Core i5 processor, 4 GB of RAM, and the Windows 10 Professional 64-bit operating system^69^.

## Results and discussion

This section introduces Exploratory Data Analysis for the ventilator dataset we used and the LSTM- ChoA model results. A complete model evaluation and comparison are presented in “Experimental simulation configuration”.

**Figure 6:**
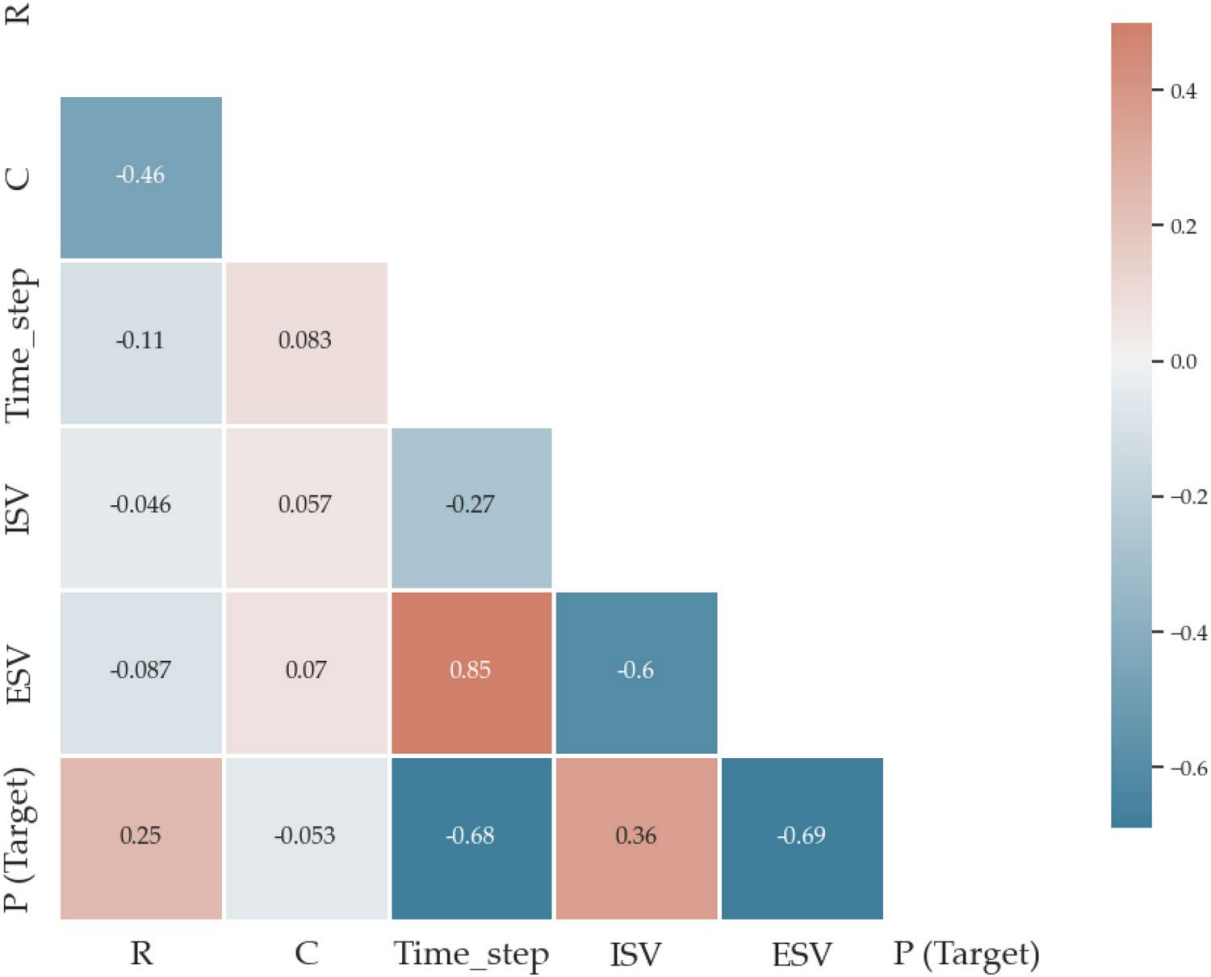
Pearson correlation matrix for dataset features.

Figure 6 displays the correlation matrix, illustrating the correlation coefficients among the different elements of interest. A value of -1 denotes a high negative correlation, while a value of 1 denotes a strong positive correlation, and the matrix determines the linear correlation between variables. The diagonal values represent the dependence of each variable on itself, also known as autocorrelation.

The correlation matrix of features indicates that there is a relatively stronger correlation between airway pressure and both features (ISV and Time step) compared to the other variables. It can be explained through the principle of the ventilator. When the value of ISV changes, the air pressure will change in the patient's bronchi, corresponding to the value of the feature "Pressure" in the dataset.

Besides airway pressure, property C also has a degree of dependency significantly with ISV. When we increase or decrease the value of the inspiratory electronic valve, the degree of lung expansion will also change (varying with the volume of air transmitted into the lungs). The remaining features have a low correlation with each other.

**Figure 7:**
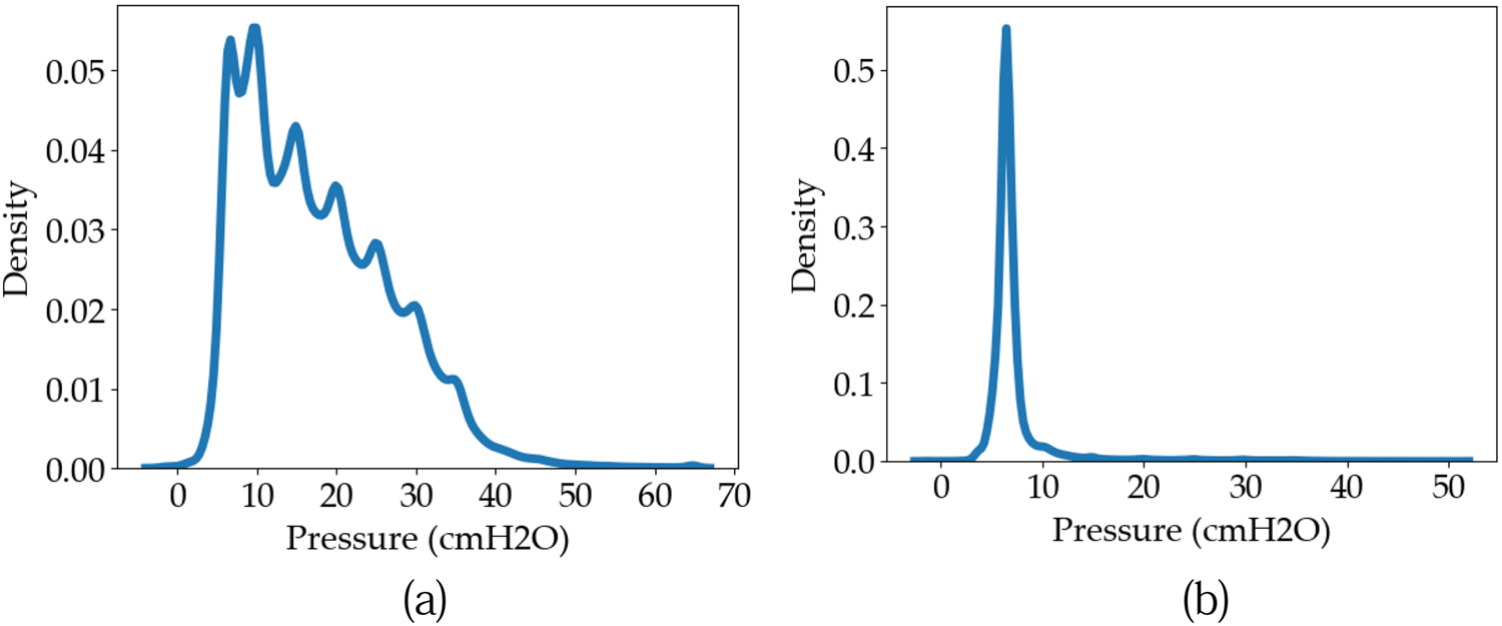
Distribution of airway pressure density. (**a**) Expiratory valve closed. (**b**) Expiratory valve open.

Figure 7 shows the pressure magnitude distributions when the expiratory valve is closed and open. From this figure, we can see that when the expiratory valve is open, the pressure distribution is left-skewed, meaning that the airway pressure lies in the lower ranges in such a case. On the other hand, when the valve is closed (referred to as the inhalation phase), the pressure lies in a wider range, which is expected as pressure variations occur during inhalation. Furthermore, notice the abrupt peaks in the distribution when the expiratory valve is closed (during inhalation), which produces small peaks in the overall distribution. The reason for these small peaks represents a slight EIV valve leak.

**Figure 8:**
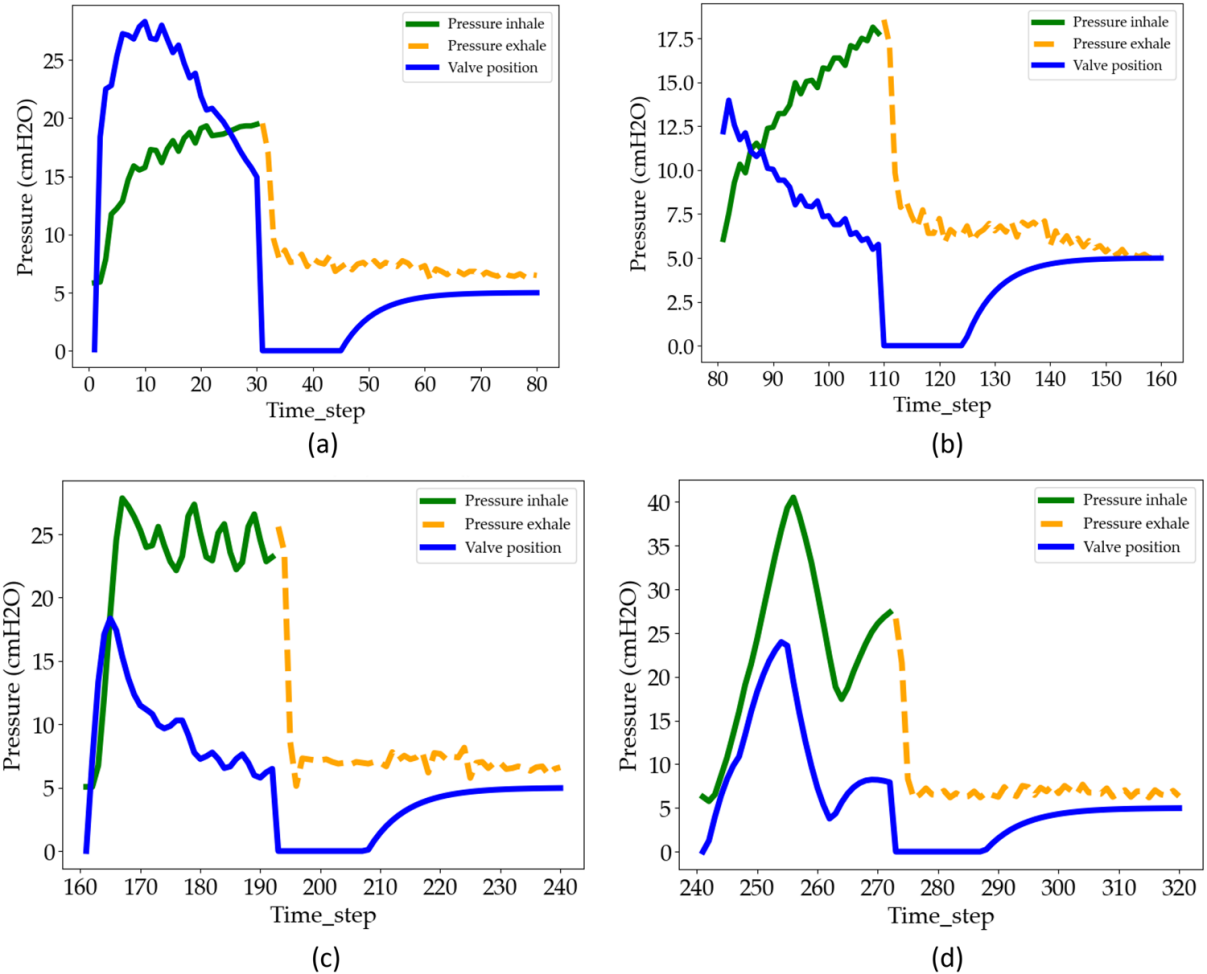
Variation of pressure and input valves position during (**a**) breadth 1, (**b**) breadth 2, (**c**) breadth 3, (**d**) breadth 4.

To illustrate how the airway pressure changes within a complete breath cycle. Figure 8 shows the airway pressure and input valve positions (opened/closed) for four different breaths. Each breath represents 80 recorded time steps.

During a breath cycle, when the expiratory valve is closed (inhalation), the pressure gradually increases as the inspiratory valve open percentage increases. Interesting to note, however, is that there is a certain ‘delay time’ between the change of valve position (control variable) and the pressure (response). Once the pressure reaches the peak value, the expiratory valve is opened as the exhalation phase begins. The pressure decreases rapidly until it reaches an asymptote. This cycle repeats continuously. It understands from this that we realize that pressure at consecutive time steps bears strong correlations with each other, meaning that sequential treatment of the data can prove advantageous.

**Figure 9:**
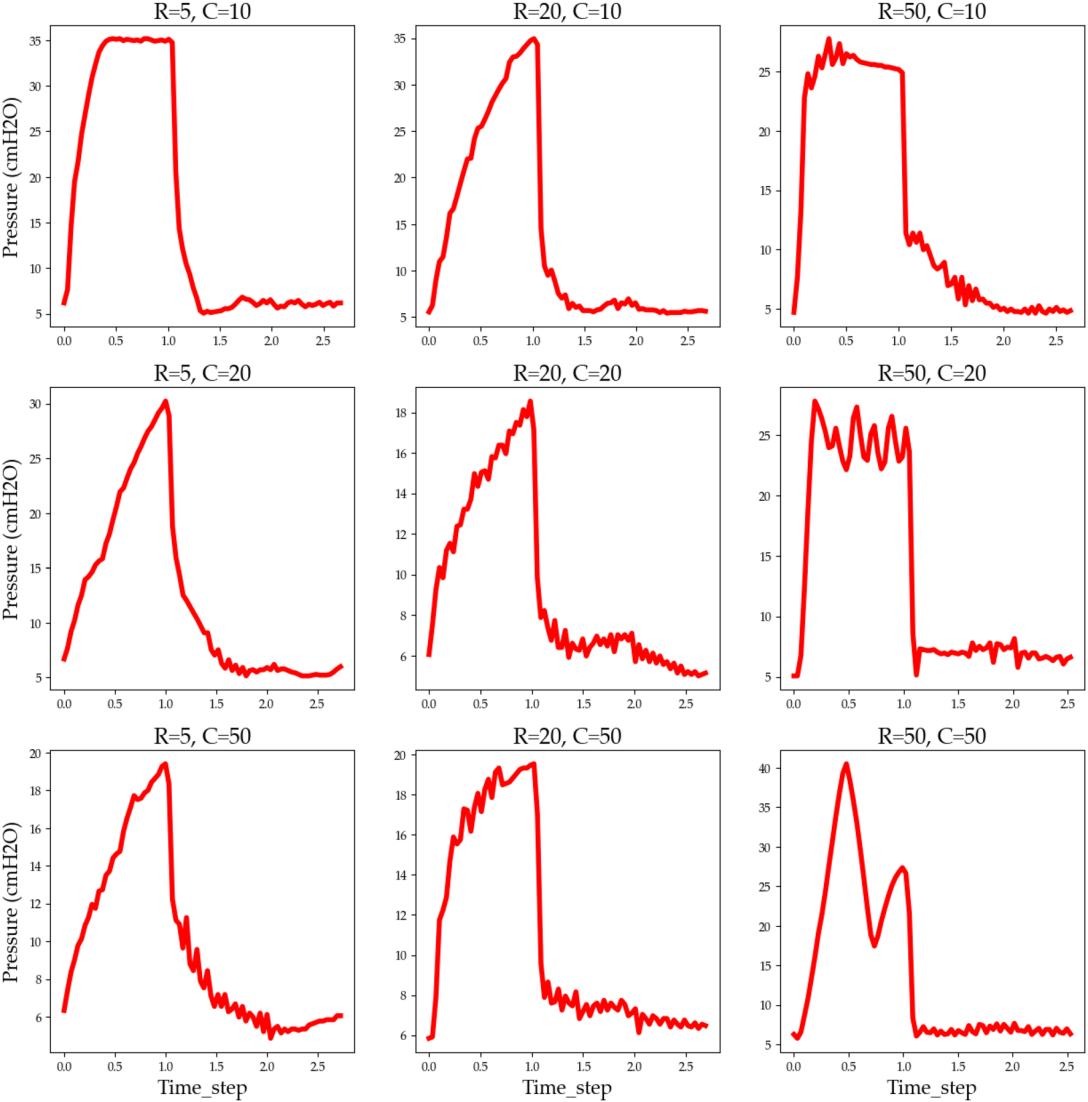
Airway pressure distribution with various lung setting.

Figure 9 shows the effects of R–C pairs on airway pressure distribution. It has nine combinations of R and C. It has nine varieties of experiments; C can be 10, 20, or 50, and R can be 5, 20, or 50. It is observed that, with different R–C pairs, the pressure distribution varies for the output valve closed and open. Therefore the adding R–C pair as a new feature in the training dataset may have a positive impact on the LSTM- ChoA model's ability to predict ventilator pressure.

**Figure 10:**
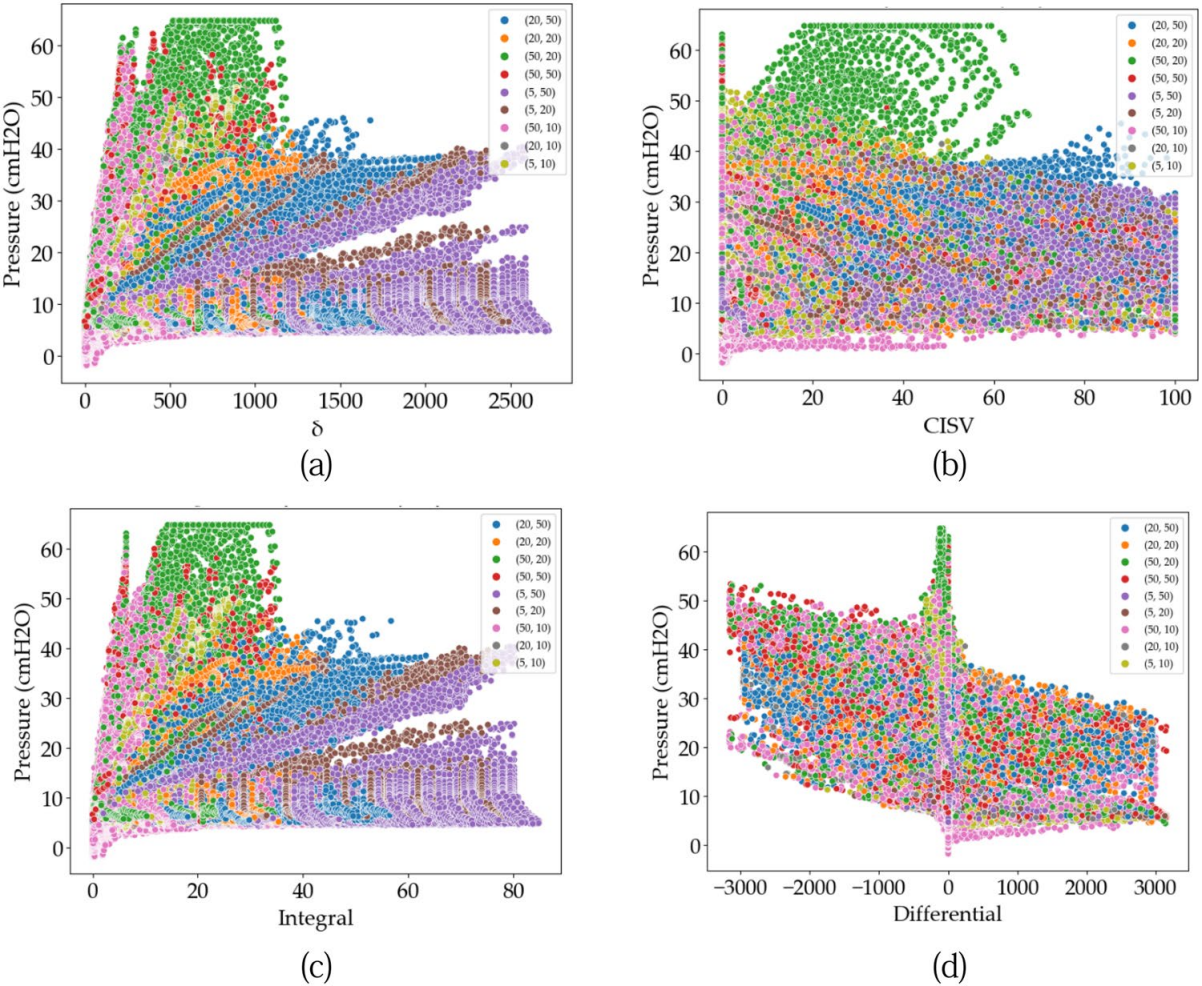
Airway pressure distribution based on extracted features (**a**) δ, (**b**) CISV, (**c**) integral, and (**d**) differential.

This study has extracted four features (δ, CISV, Integral, and Differential) to improve the model predictions. Figure 10 shows the airway pressure distribution based on extracted features and nine R-C combinations.

It is observed in Fig. 10a–c that the air pressure variation will appear and certain distinct clusters are formed. At the same time, in Fig. 10d, the differential feature fails to discriminate our dataset into certain clusters. Therefore, the features (δ, CISV, and Integral) will be preserved while will Differential feature will be neglected.

For the prediction of airway pressure, two experiments were conducted in this study; in the first experiment, four metaheuristic optimization models (PSO, GWO, WOA, and ChoA) were applied to find an efficient LSTM hyperparameter (N_I_, L_R_, N_HU,_ and N_L_) can better improve the model accuracy. In the second experiment, the prediction efficiency of LSTM-ChoA was compared with other regressor models. In two experiments, the training set accounts for 80% of the entire ventilator dataset and is used to establish regressor models. The remaining 20% is the test set used to measure the selected hyperparameters' performance.

Table 5 showcases the parameter values associated with LSTM and the corresponding computed errors derived from the initial experiment. The outcomes clearly illustrate that optimizing hyperparameters using ChoA can substantially enhance the regression performance of the LSTM model. This improvement is generally evident. ChoA outperformed Another Comparative optimization techniques PSO, GWO, and WOA, with the lowest MSE value (0.0852). The LSTM-PSO and LSTM-WOA have a similar value of MSE.

**Table 5:** The LSTM hyper-parameters value and prediction errors based on optimization algorithms.

Algorithm	Hyper-parameters value				MSE	CT (s)
	N_I_	L_R_	N_HU_	N_L_		
LSTM-ChoA	95	0.0852	93	25	0.0852	23.21
LSTM-PSO	86	0.2192	54	78	0.2192	13.40
LSTM-GWO	43	0.1789	96	51	0.1789	18.12
LSTM-WOA	23	0.2616	61	95	0.2616	8.58

Furthermore, the overall time required (referred to as computational time or CT) to accomplish a hyperparameter optimization procedure using fivefold cross-validation is employed as a metric for assessing model efficiency. It has been observed that the computational time of ChoA is generally lengthier compared to other optimization methods, whereas WOA consistently achieves the shortest time.

**Figure 11:**
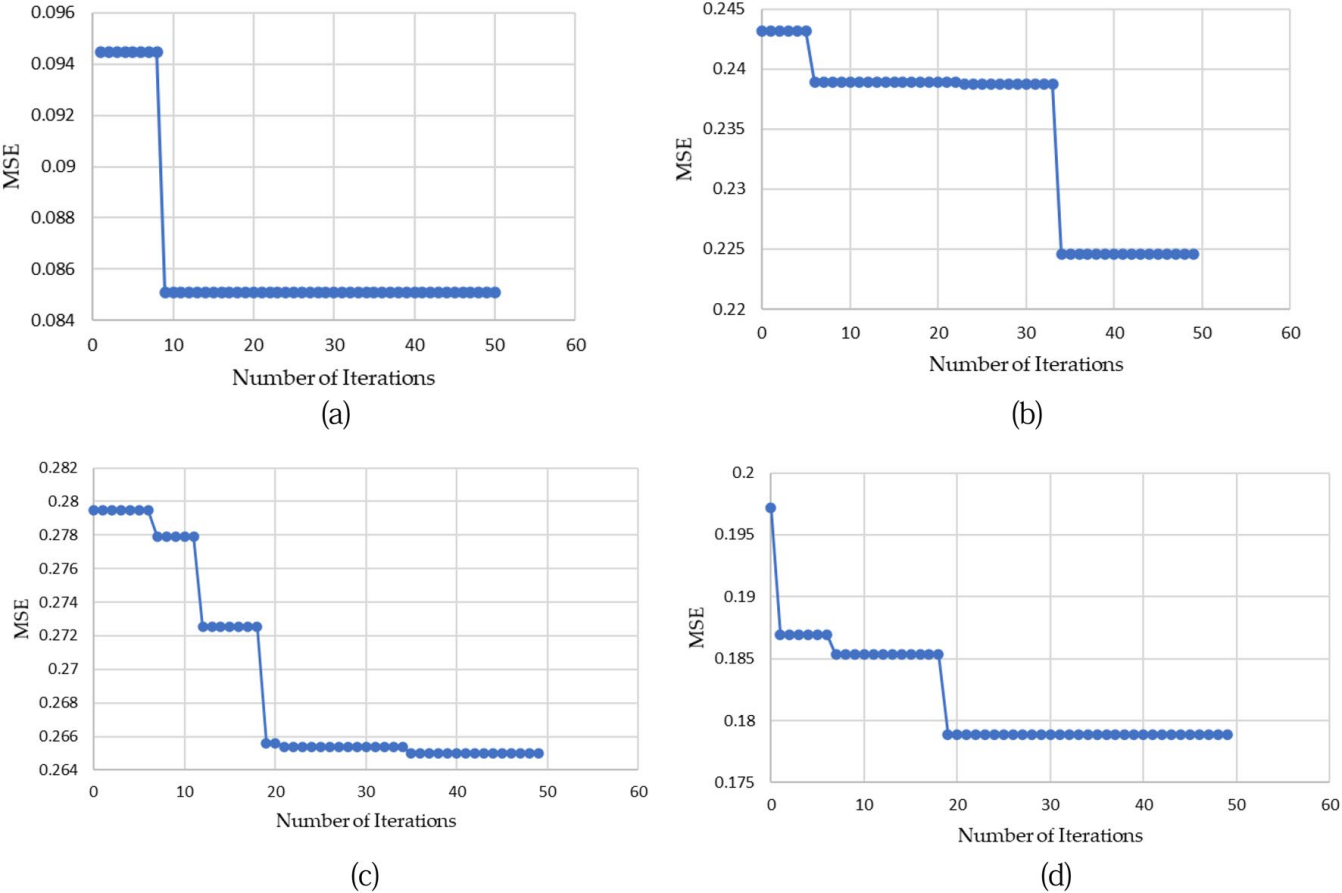
Convergence curves of the LSTM regression model based on (**a**) ChoA, (**b**) PSO, (**c**) WOA and (**d**) GWO.

Figure 11 depict the convergence curves of the LSTM model employing four distinct optimization algorithms. These figures reveal that the utilization of ChoA resulted in superior performance for the LSTM model. Specifically, the LSTM-ChoA model demonstrated better outcomes compared to other regression models, exhibiting faster and deeper convergence towards the optimal solution. Conversely, the LSTM-GWO approach also exhibited competitive performance in this study.

Considering that the regression outcome of each model is the average mean squared error (MSE) derived from 10 independent iterations, a logical step was to employ ANOVA test at a 95% confidence level. This test aimed to determine whether the classification performance achieved by the proposed LSTM-ChoA method exhibited a noteworthy superiority over other approaches. In this statistical evaluation, the null hypothesis suggested that the classification performance of distinct methods was comparable. Should the calculated p-value fall below 0.05, it would signify the rejection of the null hypothesis, indicating a significant divergence in classification performance between the methods being compared.

The outcomes of the ANOVA, along with corresponding p-values, are presented in Table 6, with LSTM-ChoA used as the benchmark method. It is evident from the results that the regression performance of LSTM-ChoA displayed a notable improvement (p-value < 0.05) across the 10 independent iterations.

**Table 6:** The result of ANOVA with p-values (the LSTM-ChoA is used as reference algorithm).

Run number	P-value		
	LSTM-PSO	LSTM-GWO	LSTM-WOA
1	0.05622	0.02442	0.04392
2	0.03632	0.03672	0.01652
3	0.01442	0.04052	0.02602
4	0.02772	0.05262	0.02342
5	0.03932	0.01382	0.05502
6	0.01362	0.05252	0.04552
7	0.05602	0.01412	0.01742
8	0.04542	0.04762	0.03472
9	0.03182	0.01432	0.02832
10	0.01672	0.01312	0.03012

In order to demonstrate the superior performance of the LSTM-ChoA prediction model, it was compared to other regression models such as RF, SVM, and KNN in the second experiment. Table 7 presents a summary of the results obtained from these predictive models in terms of mean squared error (MSE). As mentioned earlier, the hyperparameters for these models were determined using a grid search. It is evident from the table that the LSTM-ChoA model outperforms the other regression models by achieving the lowest MSE, highlighting its effectiveness in making accurate predictions.

**Table 7:** Prediction errors for comparative models.

Algorithm	MSE
RF	0.4161
SVM	0.2362
KNN	0.3142
LSTM-ChoA	0.0852

**Figure 12:**
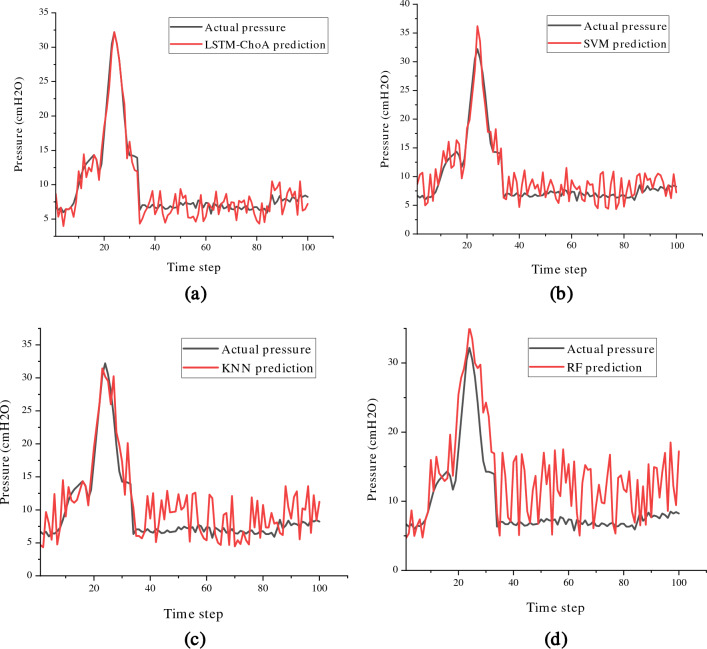
The prediction results of (**a**) LSTM-ChoA, (**b**) SVM, (**c**) KNN, and (**d**) RF.

Figure 12 displays a contrast between the factual values and the projected values for airway pressure. The figure unmistakably reveals that the prediction line generated by the RF model notably diverges from the actual pressure line, underscoring its subpar predictive performance. On the other hand, the prediction lines produced by the LSTM-ChoA model closely align with the actual lines, indicating its ability to accurately capture the underlying patterns and trends in the data.

Finally, our proposed prediction model LSTM-ChoA is compared with comparative studies^1,4,70^. They were chosen because these studies used the same dataset with different prediction techniques. As evidenced by Table 8. The Improved Chimp Optimization Algorithm has better optimization results compared to the prediction results of the other contemporary works for predicting ventilator pressure. This further demonstrates the feasibility and effectiveness of the Improved Chimp Optimization Algorithm.

**Table 8:** comparing proposed model result with comparative studies.

References	Prediction model	MSE
Alam et al.^1^	Hybrid Bi-directional -LSTM and Bi-directional gated recurrent unit (Bi-GRU)	0.145
Abdelghani Belgaid^4^	Residual network Bi-directional -LSTM (ResBiLSTM) model	0.15
Wadne et al.^70^	LSTM, RNN	0.3256
Our proposed model	LSTM-ChoA	0.0852

The LSTM model built on ChoA provides a lot of benefits. The pressure ventilator prediction forecasting accuracy significantly increased as compared to the other models. You can sum up the LSTM-ChoA model as follows:

1. The Chimp Optimization Algorithm played a vital role in optimizing the hyperparameters of the LSTM model, leading to a substantial enhancement in forecasting accuracy. This improvement is evident from the results presented in Table 4. The MSE has decreased by about 14.8% when using ChoA compared with GWO, while The MSE has decreased by about 60% ChoA compared with PSO and WOA.
2. The results in Fig. 11 have shown that both the LSTM-ChoA algorithms have the best convergence curve followed by LSTM-GWO and LSTM-PSO, LSTM-WOA at the end.
3. The ANOVA results with p-values in Table 5 show that the regression performance of LSTM-ChoA was significantly better (p-value < 0.05) on the ten independent runs.
4. The assessment of the proposed algorithm encompassed various benchmark methods including RF, SVM, and KNN. It becomes evident upon reviewing Table 6 and Fig. 12 that the LSTM-ChoA model surpasses alternative regression models, attaining the lowest mean squared error (MSE).

## Study limitation

In this study, while we have made significant progress in predicting airway pressure in ventilator respiratory circuits, it's important to acknowledge certain limitations:

1. Our study heavily relies on the ventilator pressure prediction dataset obtained from the Google Brain public dataset on Kaggle. The limitations of this dataset, such as its size, diversity, and representativeness of real-world clinical scenarios, could impact the generalizability of our findings to broader healthcare settings.
2. While ChoA helps optimize the hyperparameters of the LSTM model, the choice of hyperparameters and the tuning process is not exhaustively discussed. A more comprehensive exploration of hyperparameter sensitivity could further enhance our model's performance.
3. Although we mention feature extraction, we do not delve into the preprocessing steps in great detail. High-quality data preprocessing is crucial in building robust predictive models, and its impact on our model's performance deserves more attention.
4. Our study does not extensively discuss the generalizability of the model to other clinical settings or diverse patient populations. This raises questions about the external validity of our findings.
5. Our study does not cover whether the model's predictions have been validated in actual clinical practice. The practical applicability and safety of the model in real-world healthcare scenarios remain a subject of further investigation.

These limitations highlight areas where future research can make advancements, and they are essential for providing a more comprehensive and robust solution for airway pressure prediction in ventilator respiratory circuits.

## Conclusion

In this research paper, we made use of the ventilator pressure prediction dataset sourced from Google Brain on Kaggle. This dataset originates from an open-source ventilation system created by researchers at Princeton Lab. Our proposed approach for predicting airway pressure in ventilator respiratory circuits involves a comprehensive analysis. This entails exploratory data analysis, feature extraction, regression model creation, performance analysis, and comparison of the outcomes with reference models.

We discovered a significant relationship between airway pressure and parameters like ISV and Time step during the exploratory data analysis. The distribution of feature C also showed distinct peaks when the expiratory valve was closed, indicating a minor leak in the EIV valve, and feature C showed a considerable dependence on ISV. We retrieved four features (, CISV, Integral, and Differential) to improve the prediction model. This investigation strengthens the case for sequential data treatment's efficacy.

The LSTM Model Hyperparameters (NI, LR, NHU, and NL) were optimized using the Chimp Optimization Algorithm (ChoA). ChoA's effectiveness was assessed and contrasted with that of other benchmark optimization algorithms like PSO, GWO, and WOA. The MSE has decreased by about 14.8% when using ChoA compared with GWO, while The MSE has decreased by about 60% ChoA compared with PSO and WOA. The ANOVA results with p-values in Table 5 show that the regression performance of LSTM-ChoA was significantly better (p-value < 0.05) on the ten independent runs. Moreover, the LSTM-ChoA model was compared to regression models (RF, SVM, and KNN) as baseline models. Through extensive experimentation, ChoA demonstrated superior performance in finding the optimal LSTM Model Hyperparameters. Consequently, the LSTM-ChoA model exhibited remarkable prediction capacity and achieved the highest accuracy among all the tested models. Hence, it can be deduced that the suggested LSTM-ChoA model surpasses alternative models in terms of prediction accuracy.

The preceding overview and analysis represent the insights derived from this experiment. By comprehending the principles of the Chimp Optimization Algorithm, enhancements were made to the original LSTM algorithm, resulting in improved prediction efficiency. Consequently, future research will be valuable for exploring its application in more intricate domains, such as multi-objective optimization (Supplementary Information S1).

### Supplementary Information

Supplementary Table 1.

## Data Availability

The datasets analyzed during the current study are available on the Kaggle website, [https://www.kaggle.com/competitions/ventilator-pressure-prediction].
