# Supplementary material for: Development of a hybrid LSTM with chimp optimization algorithm for the pressure ventilator prediction
Source: Sci Rep. 2023 Nov 27;13:20927. doi: 10.1038/s41598-023-47837-8 (PMC10684522; doi:10.1038/s41598-023-47837-8)
Supplement: Supplementary file 1 — Supplementary Table 1. [file 41598_2023_47837_MOESM1_ESM.docx]

**Supplementary Table**

| **Abbreviations** | | | |
| --- | --- | --- | --- |
| LSTM | Long Short-Term Memory | SVM | Support Vector Machine |
| ChoA | Chimp Optimization Method | MSE | Mean Square Error |
| GWO | Grey Wolf Optimizer | PID | Proportional-Integral-Derivative |
| WOA | Optimization Algorithm | RNN | Recurrent Neural Network |
| PSO | Particle Swarm Optimization | GA | Genetic Algorithm |
| KNN | K-Nearest Neighbor | SA | Simulated Annealing Algorithm |
| RF | Random And Forest | ACO | Ant Colony Optimization |
| ABC | Artificial Bee Colony | HS | Harmony Search |
| DE | Differential Evolution | FA | Firefly Algorithm |
| FPA | Flower Pollination Algorithm | BA | Bat Algorithm |
| DA | Dragonfly Algorithm | MFO | Moth-Flame Optimization Algorithm |
| EWA | Earthworm Optimization Algorithm | SSA | Squirrel Search Algorithm |
| EHO | Elephant Herding Optimization | ReLU | Rectified Linear Unit |
| BSO | Brain Storm Optimization Algorithm | MS | Moth Search |
| FWA | Fireworks Algorithm | SSA | Salp Swarm Algorithm |
